# Migraine is associated with a higher risk of ischemic and hemorrhagic stroke: an analysis of the *All of Us* database

**DOI:** 10.3389/fpain.2025.1646142

**Published:** 2025-10-01

**Authors:** Nick Seah, Catherine D. Chong, Oana M. Dumitrascu, Todd J. Schwedt, Zhichao Cao, Teresa Wu

**Affiliations:** ^1^ School of Computing and Augmented Intelligence, Arizona State University, Tempe AZ, United States; ^2^ ASU-Mayo Center for Innovative Imaging, Tempe, AZ, United States; ^3^ Department of Neurology, Mayo Clinic, Phoenix, AZ, United States

**Keywords:** migraine with aura, migraine without aura, stroke, chronic migraine, risk of stroke

## Abstract

**Background:**

While prior studies suggest an increased risk of stroke among individuals with migraine, particularly those with migraine with aura, data on how specific migraine characteristics and comorbidities influence this risk across diverse populations remain limited. The *All of Us* database provides a unique opportunity to address this gap given its large sample size and inclusion of historically underrepresented groups.

**Methods:**

A cross-sectional case-control analysis using multivariable regression models accounting for vascular risk factors and comorbidities was performed to compare the risk of stroke between individuals with and without migraine, odds ratios (OR) using a 95% confidence interval (CI) were calculated.

**Results:**

Within the *All of Us* database, 31,444 individuals received a migraine diagnosis [female = 25,374/81%, male = 5,391/17%, other = 679/2%; mean (std) age = 54.9 (15.6)] and 379,283 did not have a migraine diagnosis [female = 222,104/59%, male = 149,182/39%, other = 7,997/2%; mean (SD) age = 55.9 (17.2)]. The migraine group had a greater proportion of women (81% vs. 59%), a higher proportion of white individuals (61% vs. 55%) and fewer African American individuals (15% vs. 19%). Compared to the non-migraine group, individuals with migraine had higher rates of comorbidities, including depression (46% vs. 12%), diabetes (16% vs. 7%), tobacco use (36% vs. 15%), hyperlipidemia (52% vs. 24%), hypertension (54% vs. 26%), and atrial fibrillation (3% vs. 2%).

A multivariable regression model adjusted for differences between group demographics and comorbidities found that compared to those without migraine, individuals with migraine had a higher risk of overall stroke [OR 1.97, 95% CI (1.88, 2.07)], ischemic stroke [OR 1.38, 95% CI (1.24, 1.53)] and hemorrhagic stroke [OR 1.75, 95% CI (1.60, 1.92)]. Individuals with chronic migraine had a higher risk of overall stroke compared to the non-migraine group [OR 2.56, 95% CI ( 2.32, 2.84)] and compared to episodic migraine [OR 1.90, 95% CI (1.81, 2.00)]. Those with migraine with aura had a higher risk of stroke compared to individuals with migraine without aura [OR 1.33, 95% CI (1.20, 1.48)].

**Conclusions:**

Individuals with migraine, particularly those with chronic migraine had a higher risk of stroke compared to those without migraine and compared to individuals with episodic migraine. The risk of stroke was higher in those with migraine with aura compared to those with migraine without aura even after adjusting for vascular comorbidities. Our analysis, using data from the *All of Us* database, confirms previous findings and suggests that while vascular comorbidities are more prevalent in those with migraine, they do not fully account for the increased risk of stroke.

## Introduction

Prior studies, reviews and meta-analyses (1–4) suggest that migraine, in particular migraine with aura, is associated with an increased risk of stroke, including both ischemic and hemorrhagic subtypes (5–11). However, data on migraine characteristics, such as headache frequency (chronic migraine, episodic migraine) and the presence of aura, that might contribute to the risk of stroke are sparse. Additionally, much of the existing literature has focused on relatively homogenous populations, limiting the generalizability of findings to individuals from diverse racial, ethnic and socioeconomic backgrounds.

In this study, we interrogate electronic health record data from the *All of Us* research program which includes over 400,000 participants with approximately 80% of those originating from underrepresented populations. As such, it is one of the most diverse health databases in history and includes participants from demographic groups that are typically underrepresented in human health research (12). We aim to assess the risk of stroke in individuals with migraine compared to non-migraine individuals using the diverse data available from the *All of Us* database and to assess whether migraine-specific characteristics including aura and headache frequency influences the risk of stroke in those with migraine. Furthermore, we adjusted for multiple vascular comorbidities including hypertension, diabetes, atrial fibrillation, hyperlipidemia, tobacco use, and depression, to better isolate the contribution of migraine characteristics themselves. The goal of this study is to assess the association between migraine and stroke risk, in particular ischemic and hemorrhagic subtypes and to assess whether migraine-specific characteristics such as aura and headache frequency are independently associated with stroke risk after adjusting for known vascular comorbidities. Using the rich demographic and clinical data within the All of Us database, we aim to validate previously reported associations between migraine and stroke risk, and to determine whether these relationships persist using a more diverse and representative dataset.

## Methods

### Description of *All of Us*

The *All of Us* research program, was launched in 2018 (13, 14) and continues enrollment as of February 2025. The objective of this program is to develop a large and diverse health data repository that reflects the broad range of health outcomes across different populations. By capturing data from historically underrepresented groups, the *All of Us* program aims to improve personalized treatment and address disparities in healthcare. The database includes a wide range of data types, including electronic health record (EHR), genomic information, lifestyle data, and patient-reported outcomes. This cross-sectional case-control study was submitted for Institutional Review Board (IRB) review at Mayo Clinic and was determined to be exempt from full review. IRB approval is not required for studies using the *All of Us* Researcher Workbench, as it operates under a data passport model that allows authorized users to conduct research without requiring separate IRB review for each project.

### Study population

This study included EHR data from the *All of Us* Research Program. The *All of Us* Controlled Tier database v7 was queried on 7/12/24, which served as the study search, or baseline date.

Migraine and stroke diagnosis, comorbid conditions and cardiovascular risk factors were based on ICD9 and ICD10 diagnosis codes, as shown in Table 1. People with migraine who had more than one diagnosis code for migraine were classified as follows: (1) if an individual had a diagnosis of migraine with aura at any time prior to the search date, they were classified as having migraine with aura; (2) if an individual had a diagnosis of chronic migraine at any time prior to the search date, they were classified as having chronic migraine; (3) individuals were classified as episodic migraine if they never had a diagnosis of chronic migraine. Based on these criteria, individuals were classified as either having episodic migraine with or without aura, or as having chronic migraine with or without aura.

**Table 1:** ICD9 and ICD10 diagnosis codes for (a) migraine, (b) stroke and (c) selected comorbidities.

a. Migraine		
Description	ICD9	ICD10
Migraine with aura	346.0	G43.1
Migraine without aura	346.1	G43.0
Menstrual migraine	346.4	G43.83
Chronic migraine without aura	346.7	G43.7
Other forms of migraine	346.8	G43.8
Migraine, unspecified	346.9	G43.9
b. Stroke		
Description Stroke classification	ICD9	ICD10
Acute, but ill-defined, cerebrovascular disease Ill-Defined	436	
Cerebral infarction Ischemic		I63
Occlusion and stenosis of precerebral arteries Ischemic	433	
Occlusion of cerebral arteries Ischemic	434	
Intracerebral hemorrhage Hemorrhagic	431	
Nontraumatic intracerebral hemorrhage Hemorrhagic		I61
Nontraumatic subarachnoid hemorrhage Hemorrhagic		I60
Oher and unspecified intracranial hemorrhage Hemorrhagic	432	
Oher and unspecified nontraumatic intracranial hemorrhage Hemorrhagic		I62
Subarachnoid hemorrhage Hemorrhagic	430	
c. Comorbidities		
Description	ICD9	ICD10
Atrial fibrillation	427.31	148
Depression	296, 311	F32, F33
Diabetes melitus	250, 249	E08, E09, E10, E11, E12, E13
Tobacco use disorder	V15, 305.1	F17, Z71.6, Z72.0
Dyslipidemia	272	E78
Arterial hypertension	401, 405, 642	I10, I15, O10, O11, O13, O16

### Data sources and statistical analyses

Data for this study was accessed and analyzed within the secure *All of Us* Researcher Workbench, a cloud-based platform provided by the *All of Us* Research Program. All analyses were performed in the integrated Jupyter Notebook environment, which facilitates interactive data exploration and code execution. Python (3.10.12) was employed as the primary programming language, given its extensive library ecosystem for data analysis and machine learning. Specifically, pandas (2.0.3) was used for data cleaning and preprocessing, NumPy (1.26.4) for numerical computations, and SciPy (1.11.2) for performing statistical analyses. The study involved case-control identification, data filtering, and exploratory data analysis, focusing on migraine and stroke-related variables. The final dataset contained complete data for demographic variables and diagnosis codes, with no missing values.

Continuous data distributions were assessed using both visual inspection and formal statistical testing. Age distributions for migraine and non-migraine groups exhibited deviations from normality (Shapiro–Wilk, *p* < 0.05), due to the large sample size. Nonetheless, among the candidate distributions tested (normal, exponential, gamma, and log-normal), the normal distribution offered the best overall fit and was therefore used for subsequent analyses. The percentages of individuals with and without migraine were compared using Chi-square test or independent t-test, as appropriate.

To evaluate the association between migraine and stroke risk, unadjusted and adjusted odds ratios (OR) were calculated using multivariable logistic regression which provides robustness in modeling binary outcomes and effectively captures the log odds of events. This method is particularly well-suited for scenarios where the outcome of interest, such as stroke, is rare. In such cases, ORs closely approximate risk ratios (RRs), offering a practical and interpretable measure of association. Additionally, logistic regression allows for comprehensive adjustment of multiple confounding variables.

This approach enabled the assessment of stroke risk attributable to migraine both before and after adjusting for established stroke risk factors, including age, sex at birth, hypertension, atrial fibrillation, diabetes mellitus, tobacco use, depression, and hyperlipidemia. Logistic regression models were checked for multicollinearity using Variance Inflation Factors (VIF), with all predictor variables showing VIF values below 10, indicating no multicollinearity concerns. Additionally, independence of residuals was assessed using the Durbin-Watson statistic, which was approximately 2 across all models, confirming the absence of autocorrelation. Model fit and predictive performance were evaluated using receiver operating characteristic (ROC) curves, with area under the curve (AUC) values ranging from moderate to very high (approximately 0.76–0.91), demonstrating good to very high discriminative ability (see Supplementary Material, Logistic Regression). To assess the robustness of our findings to potential misclassification of EHR data, we conducted a series of sensitivity analyses. We simulated varying degrees of exposure and outcome misclassification, including 10% underdiagnosis of migraine, 5% false positives among controls, and 5% random misclassification of both migraine and stroke diagnoses. We also modeled 5% stroke underdiagnosis and conducted a joint probabilistic bias analysis (PBA), applying 10,000 simulations based on plausible Beta-distributed values for sensitivity and specificity of both exposure and outcome. Across all scenarios in our sensitivity analyses, the association between migraine and stroke remained statistically significant. Notably, the PBA yielded an adjusted odds ratio of 2.51 (95% CI: 2.27–2.85), suggesting that diagnostic error may lead to underestimation of the true association. These findings support the robustness of our results under plausible real-world biases (see Supplementary Material, Sensitivity Analyses).

## Results

Out of a total of 410,727 people in the *All of Us* database, we identified 31,444 individuals (7.7%) with a diagnosis of migraine (migraine group). 379,283 individuals (92.3%) did not have a migraine diagnosis (non-migraine group). The migraine group had significantly greater female (81% vs. 59%) and the same amount of non-Hispanic participants (78%) (Table 2). In the migraine group, 61.5% were White, 21.9% Other, 14.6% Black or African, 1.4% Asian, 0.4% Middle Eastern or North African, and 0.1% Native Hawaiian or Pacific Islander. In the non-migraine group, 54.9% were White, 21.4% Other, 19.4% Black or African, 3.6% Asian, 0.6% Middle Eastern or North African, and 0.1% Native Hawaiian or Pacific Islander.

**Table 2:** Baseline characteristics and comorbidities of individuals with and without migraine from *All of Us* database, queried on 7/12/2024. *P*-values indicate differences in the population proportions.

Variable	Individuals with migraine	Individuals without migraine	*p*-value
*N*	31,444	379,283	
Age [mean (SD)]	54.9 (15.6)	55.9 (17.2)	<0.001 ^a^
Mean age (%) - (Overall stroke %)			
18–38	5,658 (20%) - (3.16%)	79,113 (22%) - (0.33%)	<0.001 ^b^
40–60	12,777 (41%) - (6.74%)	123,756 (33%) - (1.46%)	<0.001 ^b^
61+	13,009 (39%) - (15.95%)	176,414 (45%) - (5.41%)	<0.001 ^b^
Sex at birth [*N* (%) - (Overall stroke %)]			
Male	5,391 (17%) - (13.43%)	149,182 (39%) - (3.97%)	<0.001 ^b^
Female	25,374 (81%) - (9.16%)	222,104 (59%) - (2.45%)	<0.001 ^b^
Other	679 (2%) - (10.46%)	7,997 (2%) - (3.33%)	<0.001 ^b^
Ethnicity [*N* (%) - (Overall stroke %)]			
Hispanic or Latino	5,767 (18%) - (10.16%)	67,925 (18%) - (2.57%)	<0.001 ^b^
Non-Hispanic or Latino	24,384 (78%) - (9.76%)	296,598 (78%) - (3.16%)	<0.001 ^b^
Other	1,293 (4%) - (11.60%)	14,760 (4%) - (3.36%)	<0.001 ^b^
Race [*N* (%) - (Overall stroke %)]			
Asian	450 (1.43%) - (7.56%)	13,739 (3.62%) - (1.43%)	<0.001 ^b^
Black or African American	4,605 (14.65%) - (13.31%)	73,430 (19.36%) - (3.66%)	<0.001 ^b^
Middle Eastern or North African	141 (0.45%) - (3.55%)	2,354 (0.62%) - (1.87%)	0.279 ^b^
Native Hawaiian or Pacific Islander	35 (0.11%) - (5.71%)	447 (0.12%) - (2.91%)	0.678 ^b^
Other	6,884 (21.89%) - (10.40%)	81,164 (21.40%) - (2.73%)	<0.001 ^b^
White	19,329 (61.47%) - (9.03%)	208,149 (54.88%) - (3.11%)	<0.001 ^b^
Comorbidities [*N* (%) - (Overall stroke %)]			
Tobacco use	11,269 (36%) - (14.05%)	55,099 (15%) - (9.32%)	<0.001 ^b^
Hyperlipidemia	16,501 (52%) - (15.13%)	91,296 (24%) - (10.25%)	<0.001 ^b^
Hypertension	17,040 (54%) - (15.16%)	99,146 (26%) - (9.99%)	<0.001 ^b^
Atrial fibrillation	1,091 (3%) - (32.91%)	6,825 (2%) - (23.46%)	<0.001 ^b^
Depression	14,503 (46%) - (12.06%)	47,274 (12%) - (8.71%)	<0.001 ^b^
Diabetes	5,078 (16%) - (19.95%)	28,031 (7%) - (13.51%)	<0.001 ^b^
Overall stroke [*N* (%) (Stroke %)]	3,115 (9.91%)	11,620 (3.06%)	<0.001 ^b^
Ischemic stroke	2,526 (8.03%)	9,528 (2.51%)	<0.001 ^b^
Hemorrhagic stroke	701 (2.23%)	2,682 (0.71%)	<0.001 ^b^
Ill-Defined stroke	458 (1.46%)	1,154 (0.30%)	<0.001 ^b^
Aura status [*N* (%) - (Overall stroke %)]			
Migraine with aura	7,867 (25%) - (12.00%)		
Migraine without aura	8,797 (28%) - (8.76%)		
Other/Unspecified migraine	14,780 (47%) - (9.47%)		
Chronic status [*N* (%) - (Overall stroke %)]			
Chronic migraine without aura	3,262 (10%) - (10.70%)		
Chronic migraine with aura	1,616 (5%) - (12.44%)		
Episodic migraine without aura	26,566 (85%) - (9.66%)		

^a^ Independent sample *t*-test *p*-value.

^b^ Chi-square *p*-value.

Individuals with migraine compared to the non-migraine group had a significantly higher percentage of comorbidities including tobacco use (36% vs. 15%; *p* < 0.001), hyperlipidemia (52% vs. 24%; *p* < 0.001), hypertension (54% vs. 26%; *p* < 0.001) atrial fibrillation (3% vs. 2%; *p* < 0.001), depression (46% vs. 12%; *p* < 0.001) and diabetes (16% vs. 7%; *p* < 0.001). Individuals with migraine compared to the non-migraine group had a significantly higher percentage of stroke, including overall stroke type (3,115/31,444; 9.91% vs. 11,620/379,283; 3.06%; *p* < 0.001), ischemic stroke (2,526/31,444; 8.03% vs. 9,528/379,283; 2.51%; *p* < 0.001), hemorrhagic stroke (701/31,444; 2.23% vs. 2,682/ 379,283; 0.71%; *p* < 0.001) and ill-defined stroke (458/31,444; 1.46% vs. 1,154/379,283; 0.30%; *p* < 0.001).

Without adjusting for comorbidities (Table 3a), individuals with migraine had a 3.48 higher risk of stroke [OR 3.48, 95% CI (3.34; 3.63)] compared to individuals without migraine; when stratified by stroke type, risk of ischemic stroke was 3.39 higher [OR 3.39, 95%Cl (3.24, 3.55)], hemorrhagic stroke was 3.20 higher [OR 3.20, 95%Cl (2.94, 3.48)], and ill-defined stroke was 4.84 higher [OR 4.84, 95%Cl (4.34, 5.40)]. When stratified by migraine type, individuals with chronic migraine had a 4.02 higher risk of stroke [OR 4.02, 95%CI (3.67, 4.40)] when compared to non-migraine individuals, and individuals with migraine with aura had a 1.42 higher risk of stroke compared to individuals with migraine without aura [OR 1.42, 95% Cl (1.28, 1.57)].

**Table 3:** (a) Exploratory unadjusted logistic regression analysis of migraine (and migraine type) vs. non-migraine association with stroke occurrence among participants in the *All of Us* database. (b) The association between migraine and stroke among participants in the *All of Us* database adjusted for comorbidities (hypertension, atrial fibrillation, hyperlipidemia, diabetes, tobacco use, depression and demographics (age, sex at birth).

a. Unadjusted comparisons among various migraine groups for various stroke outcomes			
Comparison	Odds ratio	CI (95%)	*p*-value
Migraine vs. non-migraine Overall stroke	3.48	3.34–3.63	<0.001
MwA vs. MwoA Overall stroke	1.42	1.28–1.57	<0.001
Chronic migraine vs. non-migraine Overall stroke	4.02	3.67–4.40	<0.001
Chronic migraine vs. episodic migraine Overall stroke	1.19	1.08–1.31	<0.001
Migraine vs. non-migraine Ischemic stroke	3.39	3.24–3.55	<0.001
Migraine vs. non-migraine Hemorrhagic stroke	3.20	2.94–3.48	<0.001
Migraine vs. non-migraine Ill-Defined stroke	4.84	4.34–5.40	<0.001
b. Comparisons among various migraine groups for stroke outcomes adjusted for comorbidities			
Migraine vs. non- migraine Overall stroke	1.97	1.88–2.07	<0.001
MwA vs. MwoA Overall stroke	1.33	1.20–1.48	<0.001
Chronic migraine vs. non-migraine Overall stroke	2.56	2.32–2.84	<0.001
Migraine vs. non-migraine Ischemic stroke	1.38	1.24–1.53	<0.001
Chronic migraine vs. Episodic migraine Overall stroke	1.90	1.81–2.00	<0.001
Migraine vs. non-migraine) Hemorrhagic stroke	1.75	1.60–1.92	<0.001
Migraine vs. non-migraine Ill-Defined stroke	2.45	2.17–2.67	<0.001

MwA, migraine with aura; MwoA, migraine without aura.

After adjusting for comorbidities (Table 3Bb), individuals with migraine had a 1.97 higher risk of stroke [OR 1.97, 95% Cl (1.88, 2.07)] compared to individuals without migraine. Individuals with chronic migraine had a 2.56 higher risk of stroke compared to those without migraine [OR 2.65, 95% Cl (2.32, 2.84)] and a 1.90 higher risk of stroke compared to individuals with episodic migraine [OR 1.90, 95%Cl (1.81, 2.00)]. Individuals with migraine with aura had a 1.33 higher risk of stroke compared to individuals with migraine without aura [OR 1.33, 95% Cl (1.20, 1.48)]. When stratified by stroke type, individuals with migraine where at higher risk for ischemic stroke [OR 1.38, 95% CI (1.24, 1.53)], hemorrhagic stroke [OR 1.75, 95%CI (1.60, 1.92)], and ill-defined stroke [OR 2.45, 95% CI (2.17, 2.67)], compared to individuals without migraine.

## Discussion

This was a retrospective case-control analysis using EHR data from the *All of Us* database from the controlled tier v7. We found that individuals with a migraine diagnosis compared to individuals without a migraine diagnosis had a higher risk of overall stroke, ischemic and hemorrhagic stroke. The stroke risk remained significantly higher even after adjusting for age, sex, and common comorbidities, such as depression, diabetes, atrial fibrillation, tobacco use, hyperlipidemia, and hypertension.

Interestingly, people with chronic migraine had the highest risk of stroke compared to both the non-migraine and the episodic migraine group, before and after adjusting for comorbidities. Similarly, after adjustment for co-morbidities, migraine with aura had a higher risk of stroke compared to migraine without aura.

Our findings are consistent with a number of large case-control and cohort studies (5, 15–20) and several meta-analyses as well as a recent review about migraine-associated pathophysiology and the impact on stroke risk (1, 4, 21, 22) demonstrating an elevated risk of stroke (ischemic and hemorrhagic) in individuals with migraine, particularly among those with migraine with aura (5, 11, 18, 23–27). For example, Kurth et al. (15, 16, 28), using prospective cohort data from the Nurses' Health Study II, reported an increased risk of stroke in women with migraine (28). Furthermore, using data from the Women's Health study, Kurth et al. (16) reported an increased risk of total and ischemic stroke in women with migraine with aura compared to women without migraine history, and a higher risk of hemorrhagic stroke in women with active migraine with aura (but not migraine without aura) compared to women without history of migraine (28). Androulakis et al. (11, 24) demonstrated that late-middle-aged individuals with migraine with visual aura had an increased risk of ischemic stroke compared to those with migraine without aura or non-migraine individuals, using data from the Atherosclerosis Risk in Communities (ARIC) study. Additionally, their results suggested that aura onset in older age (>50) but not in younger age (<50) associated with stroke risk. This association could reflect age-related differences in pathophysiological mechanisms, including cortical spreading depression or stroke-related processes that are not dependent on atherosclerosis or to diagnostic challenges such as potentially misclassifying ischemic stroke symptoms as aura in older individuals (24, 29). It is of note that some prospective studies have shown no association, or even an inverse association, between migraine, stroke, and vascular risk factors, such as atrial fibrillation (30) and one brain imaging study did not find differences in pathophysiology based on functional imaging findings of migraine attacks with and without aura (31). These discrepancies emphasize the need for more nuanced exploration of how migraine interacts with vascular risk profiles.

Using the US Nationwide Inpatient Sample (NIS) hospitalizations database, Patel et al. (20) reported a higher risk of ischemic and hemorrhagic stroke in individuals with migraine compared to those without migraine.

Similarly, Kuo et al. (19), in a large population-based cohort study using Taiwan's National Health Insurance claims database, reported an almost twofold higher risk of hemorrhagic stroke in individuals with migraine compared to non-migraine individuals, aligning with our results.

Lee et al. (32), utilizing the same database as Kuo et al., found an increased risk of ischemic stroke in individuals with migraine (with and without aura) compared to non-migraine group, however, in contrast to our findings, no significant association with hemorrhagic stroke was observed. Similarly, Gaist et al. (33) using data from the UK Health Improvement Network (THIN) database did not find a higher risk of hemorrhagic stroke in people with migraine. Monteith and colleagues (34), using data from the Northern Manhattan Study, found no overall association between migraine and stroke risk. However, they observed an increased risk of stroke in older people with migraine who were smokers, whereas no such association was found in non-smokers.

We found that individuals with a chronic migraine diagnosis where at a higher risk of stroke compared to those with episodic migraine (OR 1.90) and compared to individuals without a migraine (OR 2.56) diagnosis. These results suggest that headache frequency significantly increases the risk of stroke in those with migraine. Similar to our findings, Kurth et al. (35) reported an association between high migraine attack frequency and ischemic stroke in women, and MacClellan et al. (36) reported that women with probable migraine with aura that experienced over 12 attacks per year had an increased risk of stroke compared to women without migraine. Our findings suggest that aura and chronic migraine are independent risk factors that significantly increase the risk of stroke in individuals with migraine. These results highlight the importance of screening individuals with migraine for aura and other stroke risk factors, which may help mitigate the risk of stroke in these at-risk individuals. Although the association between chronic migraine and stroke is compelling, future studies will need to further clarity the interaction between headache chronicity and potentially modifiable risk-factors associated with stroke risk (37).

Our study has several limitations. We recognize that migraine, stroke, and migraine-related comorbidities can be derived from clinician notes, problem lists, diagnostic test results, or billing codes, yet, due to the concern that using multiple sources as outcome measures can introduce additional noise and potential bias to the data (38), we elected to focus on ICD9 and ICD10 diagnosis codes *only* for identification of these diagnoses. However, reliance on diagnostic codes from medical records may introduce measurement bias due to potential inaccuracies of migraine diagnoses. In the *All of Us* data repository, only 7.7% of individuals had a migraine diagnosis, which is lower than the one-year prevalence of 12% in the general population (39), suggesting that migraine is underdiagnosed in the *All of Us* database. Although the results indicate a significantly higher risk of ill-defined stroke in people with migraine, these findings should be interpreted with caution due to the small sample size of individuals with migraine with ill-defined stroke (*n* = 458, 1.46%). While our findings generally align with previous studies, some differences were observed. These discrepancies may be attributed to variations in how migraine and stroke were identified across studies (e.g., diagnosis codes vs. patient questionnaires). Additionally, differences in study groups, including variations in age, sex, sample size, and socio-demographic characteristics, and adjustments for confounders may also contribute to the observed differences.

## Conclusion

The results derived from the diverse and inclusive *All of Us* data repository suggest that the increased risk of stroke in individuals with migraine is only partially explained by shared comorbid conditions and suggests that migraine itself is an independent risk factor for both ischemic and hemorrhagic stroke. The increased risk of stroke amongst those who have migraine with aura and those with chronic migraine warrant further research into migraine related features (migraine with vs. without aura, headache frequency, years lived with migraine) and the underlying pathophysiological mechanisms that could contribute to this increased risk of stroke. The inclusion of individuals that are typically underrepresented in biomedical research, as captured in the *All of Us* database, contributes to the diversity of our study population and strengthens the generalizability of our results.

## Data Availability

Publicly available datasets were analyzed in this study. This data can be found here: *All of Us* - publicly available data source.
