# Supplementary material for: Migraine is associated with a higher risk of ischemic and hemorrhagic stroke: an analysis of the All of Us database
Source: Front Pain Res (Lausanne). 2025 Oct 1;6:1646142. doi: 10.3389/fpain.2025.1646142 (PMC12521163; doi:10.3389/fpain.2025.1646142)

Table 4: Logistic Regression Diagnostics

Table 4.1 Migraine vs. Non-Migraine (Overall Stroke)

Variable	Collinearity (Variance Inflation Factor)	Residual (Durbin-Watson Statistic)	Model Fit (ROC AUC)
Sex at Birth (Male)	1.05	2.00	0.88
Sex at Birth (Other)	1.02		
Migraine/Non-Migraine	1.11		
Hypertension	1.78		
Atrial Fibrillation	1.05		
Diabetes	1.23		
Tobacco Use	1.21		
Age	1.23		
Depression	1.24		
Hyperlipidemia	1.79		

Table 4.2 Migraine with Aura vs Migraine without Aura (Overall Stroke)

Variable	Collinearity (Variance Inflation Factor)	Residual (Durbin-Watson Statistic)	Model Fit (ROC AUC)
Sex at Birth (Male)	1.03	1.98	0.77
Sex at Birth (Other)	1.01		
Migraine with Aura	1.01		
Hypertension	1.33		
Atrial Fibrillation	1.04		
Diabetes	1.15		
Tobacco Use	1.08		
Age	1.42		
Depression	1.09		
Hyperlipidemia	1.47		

Table 4.3 Chronic Migraine vs Non-Migraine (Overall Stroke)

Variable	Collinearity (Variance Inflation Factor)	Residual (Durbin-Watson Statistic)	Model Fit (ROC AUC)
Sex at Birth (Male)	1.04	1.99	0.88
Sex at Birth (Other)	1.02		
Chronic Migraine	1.03		
Hypertension	1.80		
Atrial Fibrillation	1.06		
Diabetes	1.23		
Tobacco Use	1.20		
Age	1.22		
Depression	1.20		
Hyperlipidemia	1.79		

Table 4.4 Migraine vs Non-Migraine (Ischemic Stroke)

Variable	Collinearity (Variance Inflation Factor)	Residual (Durbin-Watson Statistic)	Model Fit (ROC AUC)
Sex at Birth (Male)	1.05	2.00	0.89
Sex at Birth (Other)	1.02		
Migraine/Non-Migraine	1.11		
Hypertension	1.78		
Atrial Fibrillation	1.05		
Diabetes	1.23		
Tobacco Use	1.21		
Age	1.23		
Depression	1.24		
Hyperlipidemia	1.79		

Table 4.5 Chronic Migraine vs Episodic Migraine (Overall Stroke)

**Variable**	**Collinearity (Variance Inflation Factor)**	**Residual (Durbin-Watson Statistic)**	**Model Fit (ROC AUC)**
Sex at Birth (Male)	1.03	1.99	0.76
Sex at Birth (Other)	1.01		
Chronic Migraine	1.01		
Hypertension	1.34		
Atrial Fibrillation	1.05		
Diabetes	1.16		
Tobacco Use	1.09		
Age	1.44		
Depression	1.10		
Hyperlipidemia	1.49		

Table 4.6 Migraine vs Non-Migraine (Hemorrhagic Stroke)

**Variable**	**Collinearity (Variance Inflation Factor)**	**Residual (Durbin-Watson Statistic)**	**Model Fit (ROC AUC)**
Sex at Birth (Male)	1.05	2.01	0.83
Sex at Birth (Other)	1.02		
Migraine/Non-Migraine	1.11		
Hypertension	1.78		
Atrial Fibrillation	1.05		
Diabetes	1.23		
Tobacco Use	1.21		
Age	1.23		
Depression	1.24		
Hyperlipidemia	1.79		

Table 4.7 Migraine vs Non-Migraine (Ill-Defined Stroke)

Variable	Collinearity (Variance Inflation Factor)	Residual (Durbin-Watson Statistic)	Model Fit (ROC AUC)
Sex at Birth (Male)	1.05	2.00	0.91
Sex at Birth (Other)	1.02		
Migraine/Non-Migraine	1.11		
Hypertension	1.78		
Atrial Fibrillation	1.05		
Diabetes	1.23		
Tobacco Use	1.21		
Age	1.23		
Depression	1.24		
Hyperlipidemia	1.79		

1 Supplementary Data: Sensitivity Analyses

Sensitivity Analyses of Odds ratios: Robustness of Migraine-Stroke association across misclassification scenarios

Scenario	Odds Ratio	CI Lower	CI Upper	p-Value
**Original (Observed)**	1.97	1.88	2.07	p<0.001
**10% Underdiagnosis of Migraine**	1.95	1.86	2.05	p<0.001
**5% False Positives (Migraine)**	1.70	1.63	1.77	p<0.001
**5% Random Misclassification (Migraine & Stroke)**	1.23	1.19	1.27	p<0.001
**Stroke Underdiagnosis (5%)**	1.95	1.85	2.04	p<0.001
**PBA: Combined (Migraine + Stroke)**	2.51	2.27	2.85	p<0.001

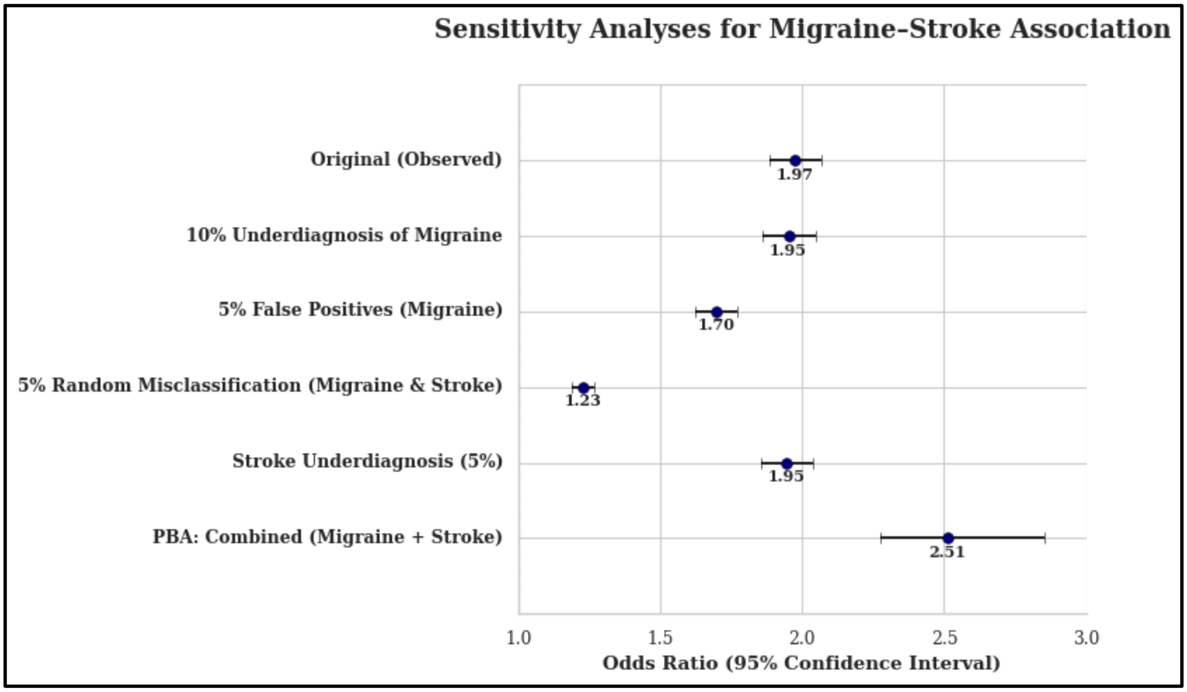

Supplement: Supplementary file 1 [file Datasheet1.pdf]
