# Supplementary material for: Migraine is associated with a higher risk of ischemic and hemorrhagic stroke: an analysis of the All of Us database
Source: Front Pain Res (Lausanne). 2025 Oct 1;6:1646142. doi: 10.3389/fpain.2025.1646142 (PMC12521163; doi:10.3389/fpain.2025.1646142)
Supplement: Supplementary file 2 [file Table1.docx]

| 1. **MIGRAINE** | | |
| --- | --- | --- |
| **Description** | **ICD9** | **ICD10** |
| Migraine with Aura | 346.0 | G43.1 |
| Migraine without Aura | 346.1 | G43.0 |
| Menstrual Migraine | 346.4 | G43.83 |
| Chronic Migraine without Aura | 346.7 | G43.7 |
| Other forms of Migraine | 346.8 | G43.8 |
| Migraine, Unspecified | 346.9 | G43.9 |
| 1. **STROKE** | | |
| **Description**  *Stroke Classification* | **ICD9** | **ICD10** |
| Acute, but ill-defined, cerebrovascular disease  *Ill-Defined* | 436 |  |
| Cerebral infarction  *Ischemic* |  | I63 |
| Occlusion and stenosis of precerebral arteries  *Ischemic* | 433 |  |
| Occlusion of cerebral arteries  *Ischemic* | 434 |  |
| Intracerebral hemorrhage  *Hemorrhagic* | 431 |  |
| Nontraumatic intracerebral hemorrhage  *Hemorrhagic* |  | I61 |
| Nontraumatic subarachnoid hemorrhage  *Hemorrhagic* |  | I60 |
| Oher and unspecified intracranial hemorrhage  *Hemorrhagic* | 432 |  |
| Oher and unspecified nontraumatic intracranial hemorrhage  *Hemorrhagic* |  | I62 |
| Subarachnoid hemorrhage  *Hemorrhagic* | 430 |  |
| 1. **COMORBIDITIES** | | |
| **Description** | **ICD9** | **ICD10** |
| Atrial Fibrillation | 427.31 | 148 |
| Depression | 296, 311 | F32, F33 |
| Diabetes melitus | 250, 249 | E08, E09, E10, E11, E12, E13 |
| Tobacco Use Disorder | V15, 305.1 | F17, Z71.6, Z72.0 |
| Dyslipidemia | 272 | E78 |
| Arterial Hypertension | 401, 405, 642 | I10, I15, O10, O11, O13, O16 |

Table 1 ICD9 and ICD10 diagnosis codes for (a) migraine, (b) stroke and (c) selected comorbidities.
