# Supplementary material for: Migraine is associated with a higher risk of ischemic and hemorrhagic stroke: an analysis of the All of Us database
Source: Front Pain Res (Lausanne). 2025 Oct 1;6:1646142. doi: 10.3389/fpain.2025.1646142 (PMC12521163; doi:10.3389/fpain.2025.1646142)
Supplement: Supplementary file 3 [file Table2.docx]

| **Variable** | **Individuals with Migraine** | **Individuals without Migraine** | **p-Value** |
| --- | --- | --- | --- |
| N | 31,444 | 379,283 |  |
| **Age (mean (SD))**  **Mean age (%) - (Overall Stroke %)**  18-38  40-60  61+ | 54.9 (15.6)  5,658 (20%) - (3.16%)  12,777 (41%) - (6.74%)  13,009 (39%) - (15.95%) | 55.9 (17.2)  79,113 (22%) - (0.33%)  123,756 (33%) - (1.46%)  176,414 (45%) - (5.41%) | <0.001^a^  < 0.001^b^  < 0.001^b^  < 0.001^b^ |
| **Sex at Birth (N (%) - (Overall Stroke %)**  Male  Female  Other | 5,391(17%) - (13.43%)  25,374 (81%) - (9.16%)  679 (2%) - (10.46%) | 149,182 (39%) - (3.97%)  222,104 (59%) - (2.45%)  7,997 (2%) - (3.33%) | < 0.001^b^  < 0.001^b^  < 0.001^b^ |
| **Ethnicity (N (%) - (Overall Stroke %)**  Hispanic or Latino  Non-Hispanic or Latino  Other | 5,767 (18%) - (10.16%)  24,384 (78%) - (9.76%)  1,293 (4%) - (11.60%) | 67,925 (18%) - (2.57%)  296,598 (78%) - (3.16%)  14,760 (4%) - (3.36%) | < 0.001^b^  < 0.001^b^  < 0.001^b^ |
| **Race (N (%) - (Overall Stroke %)**  Asian  Black or African American  Middle Eastern or North African  Native Hawaiian or Pacific Islander  Other  White | 450 (1.43%) - (7.56%)  4,605 (14.65%) - (13.31%)  141 (0.45%) - (3.55%)  35 (0.11%) - (5.71%)  6,884 (21.89%) - (10.40%)  19,329 (61.47%) - (9.03%) | 13,739 (3.62%) - (1.43%)  73,430 (19.36%) - (3.66%)  2,354 (0.62%) - (1.87%)  447 (0.12%) - (2.91%)  81,164 (21.40%) - (2.73%)  208,149 (54.88%) - (3.11%) | < 0.001^b^  < 0.001^b^  0.279^b^  0.678^b^  < 0.001^b^  < 0.001 ^b^ |
| **Comorbidities (N (%) - (Overall Stroke %)**  Tobacco Use  Hyperlipidemia  Hypertension  Atrial Fibrillation  Depression  Diabetes | 11,269 (36%) - (14.05%)  16,501 (52%) - (15.13%)  17,040 (54%) - (15.16%)  1,091 (3%) - (32.91%)  14,503 (46%) - (12.06%)  5,078 (16%) - (19.95%) | 55,099 (15%) - (9.32%)  91,296 (24%) - (10.25%)  99,146 (26%) - (9.99%)  6,825 (2%) - (23.46%)  47,274 (12%) - (8.71%)  28,031 (7%) - (13.51%) | < 0.001^b^  < 0.001^b^  < 0.001^b^  < 0.001^b^  < 0.001^b^  < 0.001^b^ |
| **Overall Stroke (N (%) (Stroke %)**  Ischemic Stroke  Hemorrhagic Stroke  Ill-Defined Stroke | 3,115 (9.91%)  2,526 (8.03%)  701 (2.23%)  458 (1.46%) | 11,620 (3.06%)  9,528 (2.51%)  2,682 (0.71%)  1,154 (0.30%) | < 0.001^b^  < 0.001^b^  < 0.001^b^  < 0.001^b^ |
| **Aura Status (N (%) - (Overall Stroke %)**  Migraine with Aura  Migraine without Aura  Other/Unspecified Migraine | 7,867 (25%) - (12.00%)  8,797 (28%) - (8.76%)  14,780 (47%) - (9.47%) |  |  |
| **Chronic Status (N (%) - (Overall Stroke %)**  Chronic Migraine without Aura  Chronic Migraine with Aura  Episodic Migraine without Aura | 3,262 (10%) - (10.70%)  1,616 (5%) - (12.44%)  26,566 (85%) - (9.66%) |  |  |

Table 2. Baseline characteristics and comorbidities of individuals with and without migraine from *All of Us* database, queried on 7/12/2024. P-values indicate differences in the population proportions.

^a^Independent sample t-test p-value

^b^Chi-square p-value
