# Supplementary material for: Migraine is associated with a higher risk of ischemic and hemorrhagic stroke: an analysis of the All of Us database
Source: Front Pain Res (Lausanne). 2025 Oct 1;6:1646142. doi: 10.3389/fpain.2025.1646142 (PMC12521163; doi:10.3389/fpain.2025.1646142)
Supplement: Supplementary file 4 [file Table3.docx]

| **Table 3a. Unadjusted comparisons among various migraine groups for various stroke outcomes** | | | |
| --- | --- | --- | --- |
| **Comparison** | **Odds Ratio** | **CI (95%)** | **p-Value** |
| **Migraine** vs Non-Migraine  *Overall Stroke* | **3.48** | **3.34 – 3.63** | <0.001 |
| **MwA** vs MwoA  Overall Stroke | **1.42** | **1.28 – 1.57** | <0.001 |
| **Chronic Migraine** vs Non-Migraine  *Overall Stroke* | **4.02** | **3.67 – 4.40** | <0.001 |
| **Chronic Migraine vs**  Episodic Migraine  *Overall Stroke* | **1.19** | **1.08-1.31** | <0.001 |
| **Migraine** vs Non-Migraine  *Ischemic Stroke* | **3.39** | **3.24 – 3.55** | <0.001 |
| **Migraine** vs Non-Migraine  *Hemorrhagic Stroke* | **3.20** | **2.94 – 3.48** | <0.001 |
| **Migraine** vs Non-Migraine  *Ill-Defined Stroke* | **4.84** | **4.34 – 5.40** | <0.001 |
| **Table 3b. Comparisons among various migraine groups for stroke outcomes adjusted for comorbidities** | | | |
| **Migraine** vs Non- Migraine  *Overall Stroke* | **1.97** | **1.88 – 2.07** | <0.001 |
| **MwA** vs MwoA  *Overall Stroke* | **1.33** | **1.20 – 1.48** | <0.001 |
| **Chronic Migraine** vs Non-Migraine  *Overall Stroke* | **2.56** | **2.32 – 2.84** | <0.001 |
| **Migraine** vs Non-Migraine  *Ischemic Stroke* | **1.38** | **1.24 – 1.53** | <0.001 |
| **Chronic Migraine vs**  Episodic Migraine  *Overall Stroke* | **1.90** | **1.81 –2.00** | <0.001 |
| **Migraine** vs Non-Migraine)  *Hemorrhagic Stroke* | **1.75** | **1.60 – 1.92** | <0.001 |
| **Migraine** vs Non-Migraine  *Ill-Defined Stroke* | **2.45** | **2.17 – 2.67** | <0.001 |

Table 3: (a) Exploratory unadjusted logistic regression analysis of migraine (and migraine type) versus non-migraine association with stroke occurrence among participants in the All of Us database. (b) The association between migraine and stroke among participants in the All of Us database adjusted for comorbidities (hypertension, atrial fibrillation, hyperlipidemia, diabetes, tobacco use, depression and demographics (age, sex at birth).

MwA= migraine with aura; MwoA= migraine without aura
